# An expanding abdominal aorta ulcer as a rare complication of *Staphylococcus aureus* sepsis in a hemodialysis patient: A case report

**DOI:** 10.1097/MD.0000000000045861

**Published:** 2025-11-28

**Authors:** Hanjing Zhou, Jun Ying, Jian Huang

**Affiliations:** a Department of Nephrology, Jinhua Central Hospital of Zhejiang university, Jinhua, Zhejiang Province, China.

**Keywords:** dialysis-related infections, hemodialysis, infected aortic ulcer, *Staphylococcus aureus*

## Abstract

**Background:**

End-stage renal disease patients on maintenance hemodialysis are at high risk of severe infections. The co-occurrence of methicillin-sensitive *Staphylococcus aureus* septicemia and penetrating aortic ulcer in this population is rare, posing notable therapeutic challenges.

**Methods:**

A case of a 73-year-old male end-stage renal disease hemodialysis patient was analyzed. The patient presented with left flank pain, nausea, and anorexia. Diagnoses and treatment adjustments were based on laboratory tests, computed tomography angiography, and serial monitoring of biomarkers and imaging.

**Results:**

Laboratory tests showed severe inflammation (white blood cell: 23.95 × 10⁹/L; C-reactive protein: 196.06 mg/L; procalcitonin: 21.15 ng/mL) and methicillin-sensitive *S. aureus* bacteremia. computed tomography angiography revealed abdominal aortic ulcer with bilateral renal artery stenosis. Initial meropenem/linezolid was switched to vancomycin post-culture; further adjustment to piperacillin-tazobactam/moxifloxacin followed ulcer progression. The patient refused surgery, received conservative treatment, and had *Candida albicans* superinfection on hospital day 20 (treated with fluconazole). He was discharged with reduced inflammatory markers and resolved symptoms, but follow-up showed ulcer expansion with hematoma (high rupture risk).

**Conclusion:**

Aortic infections in dialysis patients are therapeutically challenging. Serial biomarker monitoring and imaging are crucial for detecting disease progression, even with improved clinical symptoms.

## 1. Introduction

Patients with end-stage renal disease (ESRD) on hemodialysis are at increased risk of infections due to immune dysfunction, frequent vascular access, and comorbidities such as diabetes mellitus.^[1]^ *Staphylococcus aureus* is a common pathogen in this population, typically associated with metastatic infections including endocarditis, osteomyelitis, and abscess formation. However, aortic involvement secondary to hematogenous dissemination remains exceedingly rare. This report first presents a case of a 73-year-old male hemodialysis patient who developed an abdominal aortic ulcer secondary to *S. aureus* bacteremia. Following the case presentation, we hope it could provide evidence-based medical evidence for clinical diagnosis and treatment.

## 2. Case presentation

A 73-year-old male with ESRD on maintenance hemodialysis via a left upper arm arteriovenous fistula since 2018 presented to the emergency department with a 3-day history of left flank pain, nausea, and anorexia. He denied vomiting, diarrhea, or fever. Comorbidities included hypertension, type 2 diabetes mellitus, and hyperphosphatemia. He denies a history of coronary heart disease. He also denies a history of peripheral vascular disease, and color Doppler ultrasound of the lower extremities shows no obvious stenosis or thrombus. He had a regular hemodialysis schedule, with sessions on Tuesdays, Thursdays, and Saturdays.

**Figure 1.**
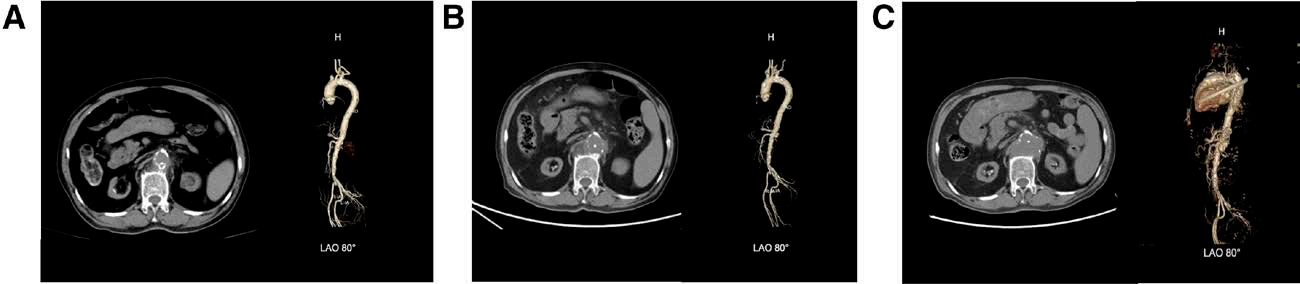
Serial CTA images showing aortic ulcer progression (A. 2024-10-10 CTA, local aortic ulceration with periaortic stranding; (B) 2024-10-22 repeat CTA, progression to penetrating ulcer expanding from 5 to 11 mm with increased inflammation; (C) 2025-01-02 Repeat CTA, multiple penetrating ulcers in the abdominal aorta with formation of a large surrounding hematoma are considered, with significant progression compared to the previous imaging). CTA = computed tomography angiography.

Initial laboratory investigations revealed a significantly elevated white blood cell count (WBC, 23.95 × 10⁹/L) with neutrophilic predominance (95.1%), along with markedly elevated C-reactive protein (CRP, 196.06 mg/L) and procalcitonin (PCT, 21.15 ng/mL), indicating a severe inflammatory response. Blood cultures obtained on admission later tested positive for *S. aureus.* Chest and abdominal computed tomography angiography demonstrated a focal ulcer in the abdominal aorta with minimal surrounding exudate, accompanied by atherosclerotic changes in the thoracic and abdominal aorta and bilateral renal artery stenosis (Fig. 1A). The patient underwent lumbar spine magnetic resonance imaging to rule out discitis, and the imaging findings showed no evidence of lumbar discitis or other spinal infectious lesions. During hospitalization, the patient was free of symptoms of chest tightness and shortness of breath. Multiple echocardiographic examinations revealed no valvular vegetations. The left ventricular ejection fraction was within the normal range, and the cardiac systolic function remained stable. No echocardiographic or clinical evidence of infective endocarditis was identified.

**Figure 2.**
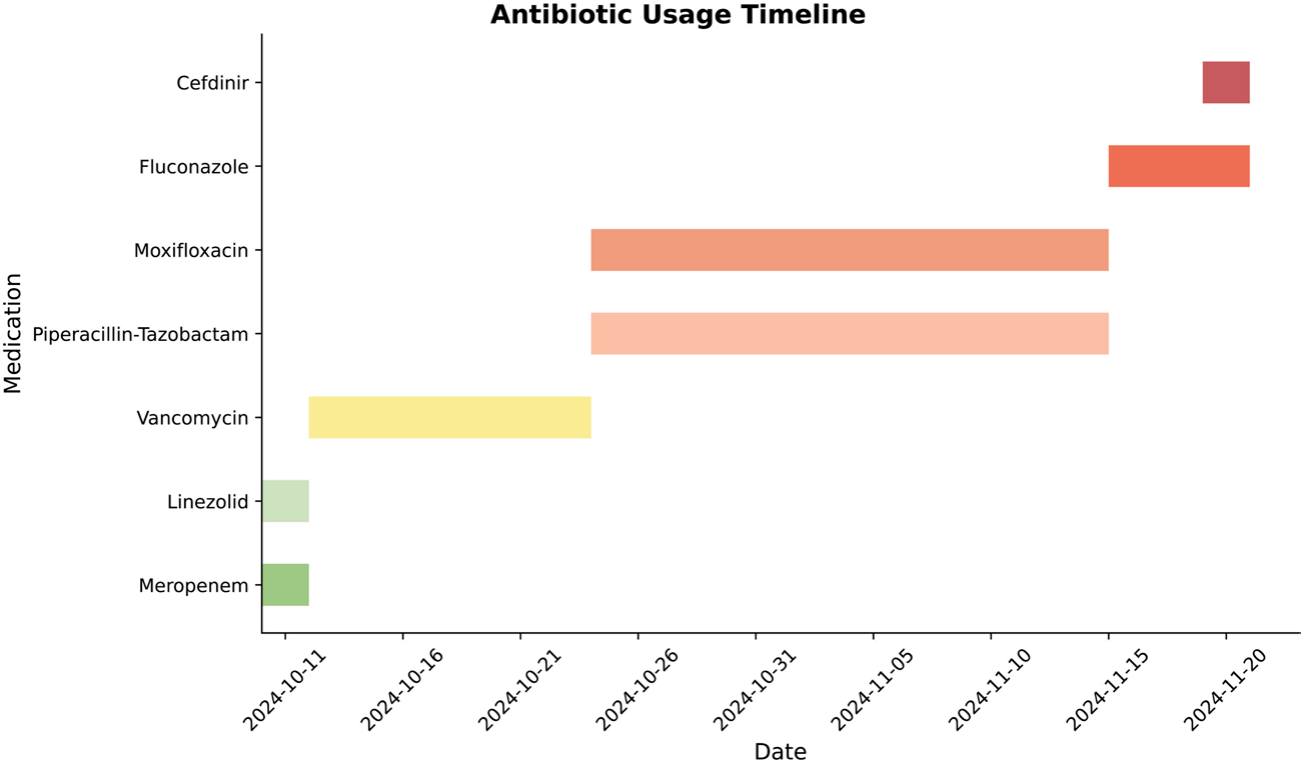
Timeline of antibiotics usage.

The patient was initially diagnosed with sepsis, uremia, and hyperkalemia, and broad-spectrum antibiotic therapy was initiated: meropenem 0.5 g intravenously every 12 hours combined with linezolid 0.6 g orally every 12 hours. Following the identification of methicillin-sensitive *S. aureus* (MSSA) in blood cultures, the regimen was switched to vancomycin 0.5 g intravenously every 8 hours. Despite initial improvement in inflammatory markers, a follow-up computed tomography angiography approximately 2 weeks later demonstrated progression of the abdominal aortic ulcer with surrounding exudative changes (Fig. 1B). The antibiotic regimen was then adjusted to piperacillin-tazobactam 4.5 g intravenously every 12 hours plus moxifloxacin 0.4 g orally daily. The timeline of antibiotic use throughout the disease course is illustrated in Figure 2.

**Figure 3.**
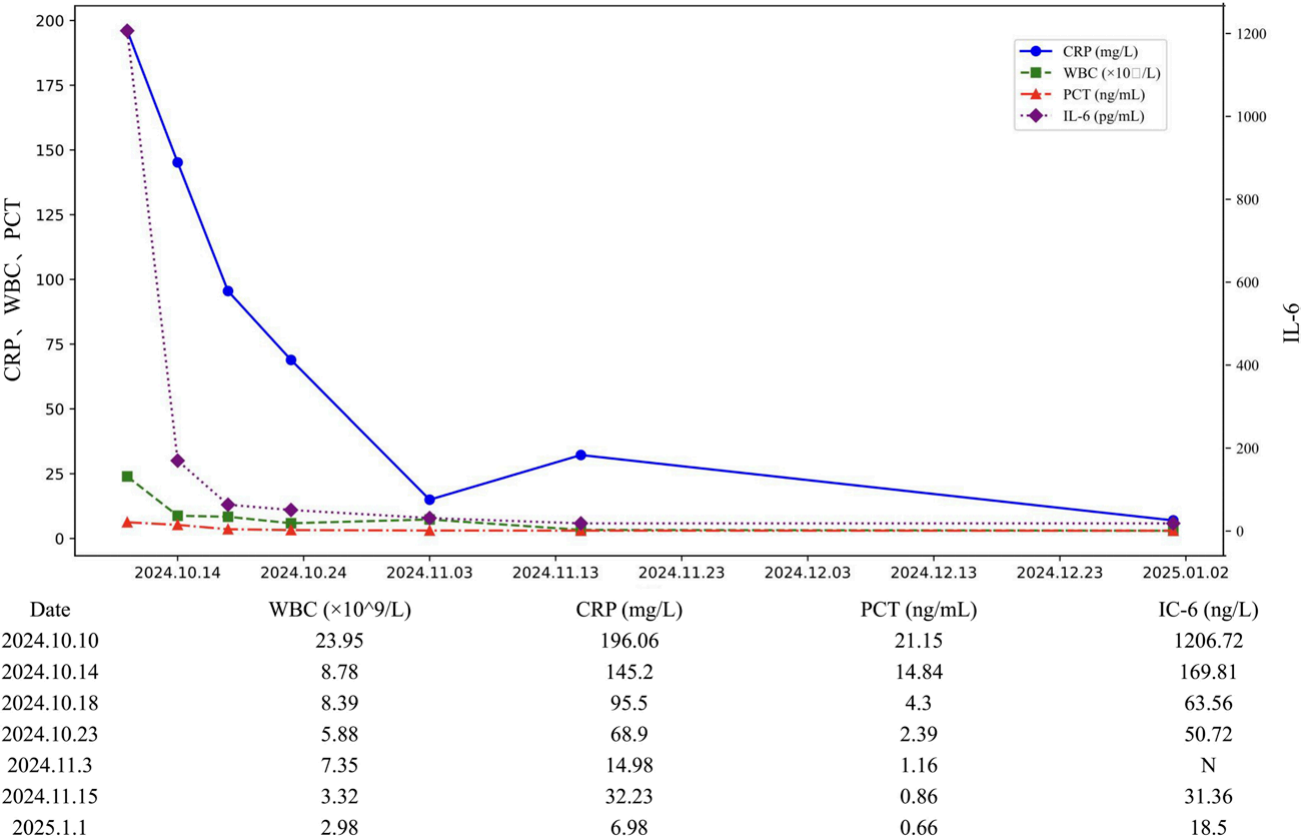
Timeline of inflammatory markers.

In view of the patient’s aortic ulcer, we promptly invited the vascular surgery department for consultation. The consultation showed that the patient had surgical indications, but after careful consideration, the patient’s family refused surgical intervention and decided to choose conservative treatment. Thereafter, the patient continued his established antibiotic regimen. On hospital day 20, blood cultures revealed *Candida albicans*. In view of this, antifungal therapy with fluconazole was added for 5 days at a loading dose of 400mg, followed by 200mg daily. After a period of treatment, the patient’s subsequent inflammatory markers showed a downward trend (see Fig. 3 for a detailed timeline of inflammatory markers). During this period, the patient’s symptoms such as low back pain and abdominal pain gradually improved. On November 21, 2024, the patient met the discharge criteria and went through the discharge procedures.

After discharge, patients were followed up as required in the outpatient clinic. At the last follow-up examination on January 2, 2025, the patient’s ulcer had progressed significantly as compared with the previous examination and was associated with a large hematoma (see Fig. 1C for details). At present, the patient’s ulcer status is extremely unstable, which may lead to hematoma rupture and bleeding at any time. Despite appropriate antibiotic therapy and a full range of supportive care, persistent abdominal aortic ulcers carry a risk of life-threatening complications, including rupture, which can mean a poor prognosis.

## 3. Discussion

This study reports an expanding abdominal aorta ulcer as a rare complication of *S. aureus* Sepsis in a hemodialysis patient. *S. aureus* is a critical pathogen in hemodialysis-associated infections. It has been reported that *S. aureus* accounts for 43.8% of bloodstream infections in hemodialysis patients, and the 30-day mortality rate is about 16%.^[2]^ Approximately 30% of MSSA bacteremia patients develop complications such as endocarditis and osteomyelitis due to bacterial dissemination,^[3]^ but research on the direct associative mechanisms with abdominal aortic ulcer is limited.

Hemodialysis patients with uremia exhibit defects in both cellular and humoral immunity, characterized by reduced neutrophil chemotaxis, impaired phagocytosis, and decreased T/B lymphocyte activity.^[4]^ In this case, the patient’s long-standing atherosclerosis led to plaque rupture, which disrupted the integrity of the aortic wall and induced ulceration – consistent with previous findings that penetrating atherosclerotic ulcers most frequently occur at sites of the most severe calcification.^[5]^ Subsequently, the local injury from the ulcer, combined with the patient’s immunodeficiency (impaired cellular and humoral immunity due to ESRD, and exacerbated immunosuppression caused by diabetes^[4]^), created conditions for the colonization and infection of MSSA, ultimately resulting in secondary infection. While the inflammatory response caused by *S. aureus* infection may accelerate the progression of aortic calcification, affect vascular wall repair and remodeling through immune imbalance, and thereby promote the occurrence and development of abdominal aortic ulcers, the specific molecular mechanisms involved require further investigation.

No clear source of infection was identified in this case. *S. aureus* infections can occur through multiple pathways in hemodialysis patients. Vascular access site infections and nasal colonization are common routes of *S. aureus* infection, while others include pulmonary infection, skin infection, and catheter-associated urinary tract infection.^[6]^ It is considered that frequent punctures of the arteriovenous fistula may have caused skin damage, and local hematoma or pseudoaneurysm may have become a hotbed for bacterial colonization.

In this case, the patient developed a mixed infection of *Candida albicans* and *S. aureus*, which may lead to increased proliferation of *S. aureus* and biofilm formation, upregulation of virulence factors, and significant increases in resistance genes to *β*-lactams, vancomycin, and azole drugs,^[7]^ thereby causing treatment difficulties. An individualized treatment plan should be formulated based on microbial culture and drug sensitivity results, combined with the patient’s specific conditions (such as age, underlying diseases, liver and kidney function), and referring to local bacterial resistance monitoring data. In recent years, the development of novel antibiotics and treatment strategies such as virulence factor-targeted therapies has provided more options.^[8]^

Without surgery, the patient in this case chose conservative treatment and was discharged after inflammatory markers decreased. However, the patient’s progressing hematoma is a potentially life-threatening condition that may rupture at any time. Evaluable surgical intervention for abdominal aortic ulcers improves long-term survival.^[9]^ According to GRADE criteria, open surgical repair is recommended for low-risk non-ruptured abdominal aortic aneurysm patients (age ≤ 80 years), while endovascular aneurysm repair is the first choice for older patients with suitable anatomy and low or high risk. If the anatomy was unsuitable for endovascular aneurysm repair, open surgical repair is performed.^[10]^ In addition, innovative treatments for abdominal aortic ulcers include BeGraft-covered stents,^[11]^ novel medium-cutoff dialysis membranes,^[12]^ and the nuclear receptor Nur77 as a potential therapeutic target.^[13]^ More evidence-based medical data are needed to support these approaches.

In addition, infection prevention is of utmost importance. In hemodialysis care, standardized measures such as chlorhexidine disinfection, aseptic technique, and infection surveillance can significantly reduce infection rates and demonstrate remarkable cost-effectiveness.^[14]^ Key strategies for central line-associated bloodstream infection prevention include antimicrobial lock solutions, minimizing the use of central venous catheters, and strict aseptic practices.^[15]^

## 4. Strengths and limitations

This study has strengths and limitations. This study documents the dynamic evolution of a complex clinical scenario in patients with ESRD, including aortic ulceration and mixed-microbial infections, with a clear timeline of associations between antibiotic modification and radiographic progression. This provides actionable insights into the individualized management of similar cases. However, the study also has limitations: as a single case report, sample bias and unmeasured patient-specific factors affecting treatment outcomes limit its generalizability. The patient’s family declined surgical intervention, so the prognostic impact could not be assessed, introducing a potential decision bias in favor of conservative management. Future studies should validate the long-term efficacy of antibiotics through cohort analysis and integrate artificial intelligence to quantify the imaging features of aortic ulcers and refine universal treatment strategies.

## 5. Conclusion

Abdominal aortic ulcer is a rare and severe complication of *S. aureus* bacteremia in hemodialysis patients. In cases of persistent bacteremia, clinicians should be aware of the potential occurrence of this complication. Early initiation of imaging examinations is crucial, followed by the timely administration of targeted antibiotic therapy. A comprehensive evaluation of the patient’s condition and comorbidities is necessary when considering surgical intervention.

## Author contributions

**Data curation:** Hanjing Zhou, Jun Ying.

**Formal analysis:** Jun Ying.

**Funding acquisition:** Hanjing Zhou.

**Resources:** Jun Ying.

**Software:** Hanjing Zhou.

**Supervision:** Jian Huang.

**Validation:** Jun Ying, Jian Huang.

**Visualization:** Jian Huang.

**Writing – original draft:** Hanjing Zhou, Jun Ying.

**Writing – review & editing:** Jian Huang.
